# Effects of Flavonoid Supplementation on Common Eye Disorders: A Systematic Review and Meta-Analysis of Clinical Trials

**DOI:** 10.3389/fnut.2021.651441

**Published:** 2021-05-25

**Authors:** Sergio Davinelli, Sawan Ali, Giovanni Scapagnini, Ciro Costagliola

**Affiliations:** Department of Medicine and Health Sciences “V. Tiberio”, University of Molise, Campobasso, Italy

**Keywords:** flavonoids, eye, supplementation, nutrition, visual impairment, ocular disorders

## Abstract

**Background:** Emerging studies show that certain plant compounds may reduce the severity of most prevalent ocular abnormalities. The aim of this systematic review and meta-analysis was to assess the effect of dietary flavonoids on major eye disorders.

**Methods:** Eligible studies were identified by searching PubMed, Web of Science, Scopus, and Cochrane Library databases for all articles published up to April 2021. The literature search yielded 1,134 articles, and a total of 16 studies were included in the systematic review. A meta-analysis of 11 intervention trials involving a total of 724 participants was performed.

**Results:** Using a random-effects model, the pooled results revealed an overall significant effect of flavonoids on common ophthalmic disorders (standard mean difference = −0.39; 95% CI: −0.56, −0.21, *p* < 0.01). Of the subclasses of flavonoids, flavan-3-ols (standard mean difference = −0.62; 95% CI: −1.03, −0.22, *p* < 0.01), and anthocyanins (standard mean difference = −0.42; 95% CI: −0.63, −0.21, *p* < 0.01) were the only effective intervention for improving the outcomes of ocular conditions. For several of the other flavonoid subclasses, evidence on efficacy was insufficient.

**Conclusion:** Our findings indicate that flavonoids may improve the clinical manifestations associated with ocular disorders. However, further well-constructed clinical trials are required to confirm these results and examine the effect of flavonoids on eye disorders other than those identified in this review.

**Systematic Review Registration:** PROSPERO, identifier CRD42021247332.

## Introduction

Ocular diseases are common in the general population, and they are major causes of reversible and irreversible blindness. Globally, of the 7.33 billion people alive in 2015, an estimated 36 million were blind, 217 million had moderate or severe vision impairment, and 188 million had mild visual impairment (1). The most common eye diseases, such as glaucoma, age-related macular degeneration (AMD), diabetic retinopathy (DR), and cataract, can cause sequelae ranging from visual impairment to irreversible blindness (2). Advancing age remains the main risk factor for several eye disorders; consequently, blindness and visual impairment are more prevalent in older adults (3). Ocular diseases cause visual impairment in 4%-20% of adults older than 65 years, depending on how impairment is defined (4). A global prevalence of 33.4% for DR, 3.54% for primary open-angle glaucoma, and 8.69% for AMD, have been estimated, particularly in people older than 60 years (5–7). Similarly, untreated cataract was the principal cause of blindness in individuals aged 50 years or older in 2015 (8). Various ophthalmic disorders are also known to adversely affect the quality of life. Poor visual function is a strong predictor of depression, anxiety, and mortality (9–12). It is therefore essential to develop more effective strategies to prevent or better manage ocular diseases.

The eye undergoes several structural changes with advancing age; these generally include the following: loss and attenuation of cells in layers such as the corneal endothelium, ganglion layer, photoreceptors, and retinal pigment epithelium (RPE); degenerative processes, such as vitreous liquefaction; and accumulation of materials, such as drusen (13–15). Recent efforts have been directed toward elucidating the biochemical mechanisms underlying common eye diseases. Mitochondrial dysfunction, decreased energy metabolism, and impaired antioxidant defenses have been reported in aging lens epithelial cells, the retina, RPE, and optic nerve (16–20). Because it is highly exposed to UV light, environmental insults, and atmospheric oxygen, the eye is vulnerable to the detrimental effects of oxidative stress. Excessive production of free radicals alters the intracellular redox state, inducing the expression of various pro-inflammatory mediators. Dysregulated inflammation and oxidative damage accelerate structural changes in ocular tissues, which facilitates the development and progression of eye pathologies that can cause vision loss (21, 22).

To date, non-pharmacological approaches focused on lifestyle interventions are considered cost-effective and practical for controlling modifiable risk factors associated with the most prevalent ocular disorders. Among the environmental factors, nutrition is critically involved in various degenerative processes linked to visual impairment (23). The contribution of certain nutrients to the prevention and management of common eye diseases has been the subject of numerous clinical trials. As summarized in recent reviews, there are human studies showing that greater adherence to healthy dietary patterns or the intake of specific nutrients (e.g., carotenoids) may improve inflammatory/oxidative alterations associated with the onset and progression of ocular diseases (24, 25).

Flavonoids are a class of polyphenolic compounds widely present in several commonly consumed fruits, vegetables, herbs, and beverages. The structural complexity of flavonoids has led to their classification into six main subclasses that include flavonols, flavones, flavanones, flavan-3-ols (including their oligomeric and polymeric forms, proanthocyanidins), isoflavones, and anthocyanins (26). Experimental studies demonstrate that dietary flavonoids and their representative subclasses interact directly with rhodopsin and modulate visual pigment function. Additional studies show that flavonoids protect various ocular cell types from oxidative stress-induced cell death, improve endogenous antioxidant systems, inhibit inflammatory pathways, and enhance mitochondrial functions (27, 28). However, clinical findings on the prevention or ameloriation of ocular diseases remain inconsistent across studies. Therefore, a systematic review and meta-analysis was conducted to critically evaluate whether flavonoids are clinically efficacious in participants affected by ophthalmic conditions.

## Methods

This systematic review and meta-analysis was conducted in accordance with established guidelines from the Preferred Reporting Items for Systematic Reviews and Meta-Analysis (PRISMA) (29). The review was registered on PROSPERO (Identifier: CRD42021247332).

### Search Strategy

A literature search strategy involving the use of controlled vocabulary (i.e., Medical Subject Headings and Index terms) and free text terms (Supplementary Table 1) was developed and used. The search terms were combined using Boolean operators “AND” and “OR” and used to search for relevant literature in the following databases: PubMed, Web of Science, Scopus, and Cochrane Library. The search was conducted to identify eligible studies published up to April 20, 2020.

### Eligibility Criteria

After the search, all the retrieved studies were assessed for eligibility by two independent authors (SD and SA) and by reviewing titles and abstracts, followed by a full-text review. Any disagreements were settled through a discussion with a third author (CC). Clinical studies that clearly assessed the effect of flavonoids on clinical outcomes associated with ocular diseases were included in the review. However, to better structure the eligibility criteria, the PICOS approach, based on the following categories, was used: (1) population; (2) intervention; (3) comparator; (4) outcome; (5) study design. Therefore, studies that fulfilled the following criteria were eligible: (1) middle-aged or older adults (≥35 years old) with a clinical diagnosis of macular degeneration, glaucoma, cataract, retinopathy, dry eye, and any other ocular conditions consistent with the search strategy; (2) intervention consisting of flavonoid supplementation (only studies that reported the flavonoid content of foods or the dose of the flavonoid-containing supplements); (3) any comparator, including no intervention; (4) effect of flavonoid supplementation on outcomes associated with ocular conditions (only studies that provided sufficient information about clinical outcomes before and after the intervention in the treated and control group); (5) clinical intervention study (any research design involving human subjects). Articles were excluded from the review for the following reasons: they were not published in English; they used secondary data from papers such as reviews, meta-analyses, conference papers, and book chapters; they were studies on animal models or *in vitro* experiments.

### Data Extraction

Data from the included studies were extracted independently by two authors (SD and SA) and cross-checked to settle any discrepancies with a third author (CC). The following data were extracted: author, year of publication, study design, type and composition of the intervention, sample sizes of the intervention and control groups, mean or range of the ages of the participants. The extracted sample size was the number of participants included for the analyses in the study (excluding participants who dropped out or were lost to follow-up). The following details were extracted for the nutritional interventions: type of intervention, dose, duration, and frequency.

### Quality Assessment

The quality assessment of studies was performed independently by two reviewers (SD and SA) using the Cochrane Risk of Bias Tool (30). Discrepancies were discussed with a third reviewer (CC). Individual quality items were examined, including random sequence generation (selection bias), allocation concealment (selection bias), blinding of participants and personnel (performance bias), blinding of outcome assessment (detection bias), incomplete outcome data (attrition bias), and selective reporting (reporting bias). Using this approach, the quality of each study was graded as high, moderate, or low based on the following criteria: (1) high quality if all domains were met (all sources of bias are low risk) or one domain was of unclear risk; (2) moderate quality if one domain was not met (high risk) and one was of unclear risk, or alternatively, if two were of unclear risk; (3) low quality if three or more domains were of unclear risk or two or more were not met (high risk).

### Statistical Analysis

Participants who consumed the flavonoid supplements were allocated to the treated group, while those who consumed the control were allocated to the control group. The summary statistics required for each outcome were the number of participants in the active and control groups post-intervention. Standardized mean differences and associated confidence intervals for the ocular outcomes of all the studies were calculated. A random-effect model was used because the interventions, participants, and assessment of outcomes differed across studies; therefore, heterogeneity was assumed (31). Heterogeneity was assessed using the *I*^2^-test and graded as follows: low heterogeneity, *I*^2^ ≤ 25%; moderate heterogeneity, *I*^2^ > 25% and ≤ 50%; high heterogeneity, *I*^2^ > 50% (32). A sensitivity test was performed by sequentially omitting one study each time to evaluate the stability of the results. Begg's funnel plots and Egger's regression test were used to investigate publication bias of the studies. Subgroup analyses were conducted according to the flavonoid subclasses and ocular conditions. Visual inspection of fthe unnel plots was used to determine publication bias. For all statistical procedures, *P* < 0.05 was considered statistically significant. All analyses were performed using R Software, version 3.2.3, and the interface R-Studio Version 0.99.491.

## Results

### Selection and Study Characteristics

**Figure 1:**
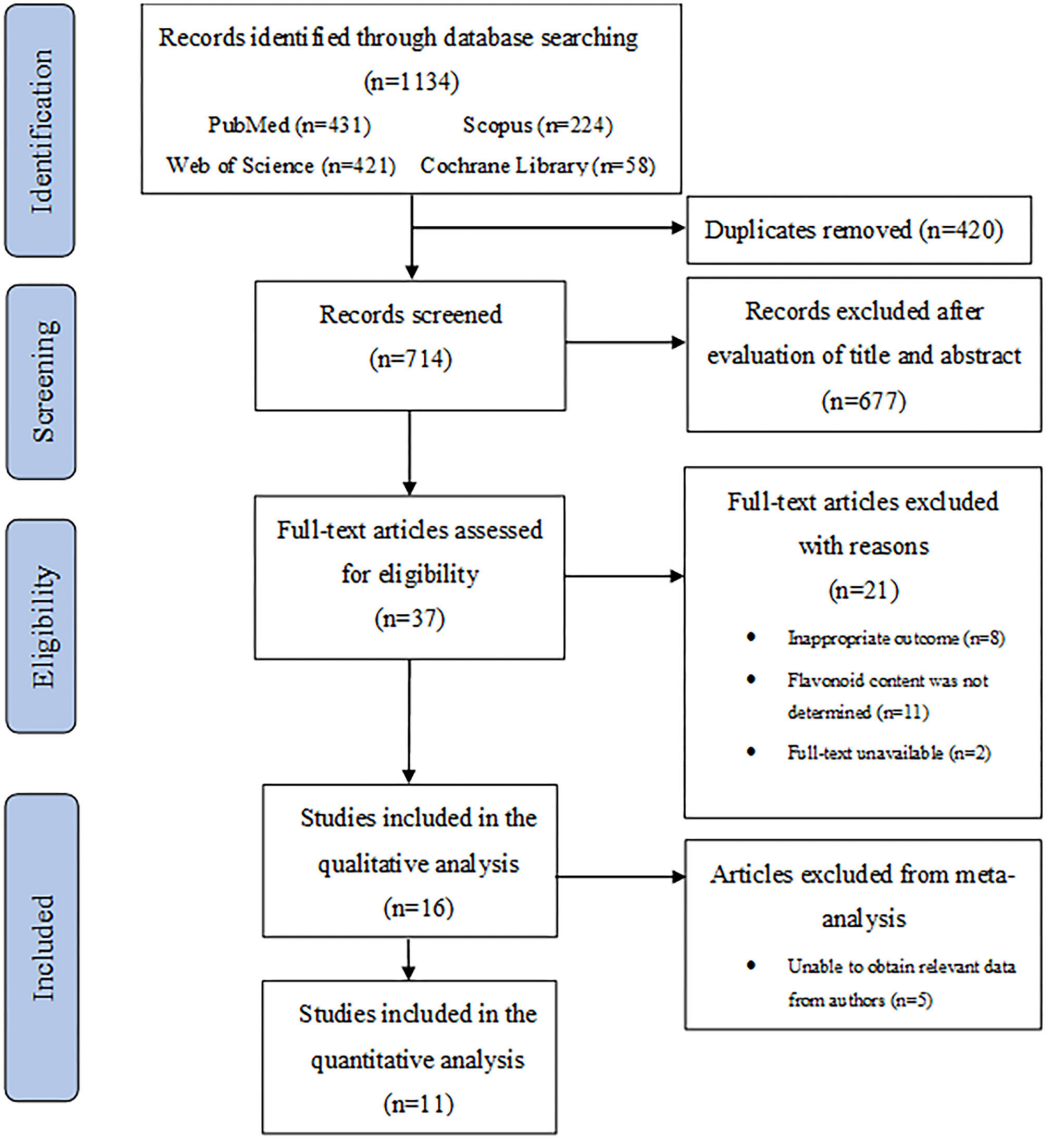
PRISMA flowchart describing a systematic literature search and study selection.

A flowchart describing the systematic search and study selection process is shown in Figure 1. The database searches yielded 1,134 records. After removing duplicates, we screened 714 titles and abstracts and identified 37 articles for full-text review. We excluded 21 studies for the following reasons: inappropriate ocular outcomes (*n* = 8); flavonoid content was not determined (*n* = 11); full-texts were unavailable (*n* = 2). Thus, 16 studies met our eligibility criteria and were included for the final analyses. Detailed characteristics of all studies included in the systematic review are described in Table 1. The studies were published between 2002 and 2021 and conducted in four different countries: Italy, Republic of Korea, Japan, and China. The mean age of participants in these studies ranged from 39 to 67 years, whereas the sample sizes ranged from 10 to 229 participants. Among the 16 included clinical studies, 11 were randomized clinical trials (RCTs) with an average number of 51 randomized participants. The remaining five clinical studies had a mean of 60 participants. All 16 studies included both male and female participants.

**Table 1:** Characteristics of studies included in the systematic review.

**References**	**Country**	**Population characteristics and study design**	**Intervention**	**Outcome of interest**	**Eye exam**	**Results**
Falsini et al. (33)	Italy	*N* = 36 (M 18 and F 18; mean age 58.9 y) Condition: Ocular hypertension and glaucoma Design: Randomized, placebo-controlled, double-blind, cross-over trial Duration: 3 months	ECGC (200 mg/d)	RGC function Visual field	PERG HFA (30–2)	In OAG patients, PERG amplitude change was greater than placebo (*p* < 0.05)
Forte et al. (40)	Italy	*N* = 40 (M 22 and F 18; mean age 62.9 y) Condition: Diabetic cystoid macular edema without macular thickening Design: Randomized controlled trial Duration: 14 months	Diosmin (300 mg/d) and Troxerutin (300 mg/d) with *Centella Asiatica* (30 mg/d) and *Melilotus* (160 mg/d)	Visual acuity Central retinal thickness Retinal sensitivity Stability of fixation	BCVA SD-OCT MP	At month 14, the retinal sensitivity was greater in the treated group than control (*p* = 0.01)
Forte et al. (41)	Italy	*N* = 70 (M 30 and F 40; mean age 64.9 y) Condition: Diabetic cystoid macular edema without macular thickening Design: Randomized controlled trial Duration: 36 months	Diosmin (300 mg/d) with *Centella Asiatica* (15 mg/d) and *Melilotus* (160 mg/d)	Visual acuity Central retinal thickness Retinal sensitivity Stability of fixation	BCVA SD-SLO/OCT HRA-OCT MP	At month 12, 24, and 36, the retinal sensitivity was greater in the treated group than control (*p* = 0.001)
Manabe et al. (42)	Japan	*N* = 18 (M 7 and F 11; mean age 57.7 y) Condition: primary open-angle glaucoma. Design: Multicentre, open-label, prospective, single-arm study. Duration: 6 weeks	Procyanidins (28 mg/d) Anthocyanins (32.4 mg/d)	IOP	IOP_GAT_ IOP_RBT_	Significant decrease in IOP_GAT_ (*p =* 0.004) and morning IOP_RBT_ (*p* = 0.029) compared to baseline
Moon et al. (34)	Republic of Korea	*N* = 66 (M 36 and F 30; mean age 59.0 y) Condition: Non-proliferative diabetic retinopathy Design: Randomized double-blind placebo-controlled multicentre trial Duration: 12 months	Grape seed proanthocyanidins (150 mg/d)	Hard exudate severity Visual acuity Central subfield mean thickness Total macular volume	FA BCVA OCT	Significant improvement in hard exudate severity (*p* < 0.05) than placebo. Significant decrease in total macular volume (*p* < 0.05) from baseline
Nebbioso et al. (43)	Italy	*N* = 32 (M 14 and F 18; mean age 56.7 y) Condition: Pre-retinopathy diabetes Design: Randomized placebo-controlled trial Duration: 1 month	Genistein (80 mg/d) with α-lipoic acid, (400 mg/d), Vitamin C (30 mg/d), Vitamin E (5 mg/d), B vitamins (15 mg/d)	Oxidative stress Electrophysiological response	Plasma antioxidant levels ERG	Significant increase of ERG oscillatory potential values in the treated group (*p* < 0.05) than control
Nebbioso et al. (44)	Italy	*N* = 10 (M 1 and F 9; mean age 67.6 y) Condition: High IOP Design: Double-blind placebo-controlled trial Duration: 6 weeks	Rutin (200 mg/d) Forskolin (15 mg/d)	IOP	GAT	Inhibition of the increase of IOP that occurs after laser iridotomy (*p* < 0.05)
Ohguro et al. (35)	Japan	*N* = 30 (M 9 and F 21; mean age 66.7 y) Condition: Normal tension glaucoma Design: Intervention trial Duration: 6 months	Anthocyanins (50 mg/d)	IOP Plasma ET-1 Retinal blood flow Visual field defects	GAT ET-1 immunoassay LDF HFA (30–2)	Significant increase in the retinal blood flow and plasma ET-1 (*p* < 0.05)
Ohguro et al. (36)	Japan	*N* = 38 (M and F; mean age 61.7 y) Condition: Glaucoma Design: Randomized double-blind placebo-controlled trial Duration: 24 months	Anthocyanins (50 mg/d)	IOP Optic nerve head examination Ocular blood circulation Visual field mean deterioration	GAT LSFG HFA (30–2)	Significant less visual field mean deterioration (*p* = 0.039) and significant improvement of ocular blood circulation (*p* = 0.01), compared with placebo
Ohguro et al. (37)	Japan	*N* = 12 (M 6 and F 6; mean age 39.4 y) Condition: High IOP Design: Double-blind placebo-controlled trial Duration: 10 weeks	Anthocyanins (50 mg/d)	IOP Visual field mean deterioration	GAT HFA (30–2)	Significant decrease in IOP (*p* < 0.05) from baseline and compared with placebo
Ren et al. (38)	China	*N* = 30 (M 17 and F 13; mean age 62.9 y) Condition: Diabetic retinopathy Design: Randomized controlled trial Duration: 6 weeks	Puerarin (400 mg/d)	Hemorrheological changes in central retinal artery and vein	PSV EDV A CRVRV	Significant improvement in PSV, EDV, A, and CRVRV values (*p* < 0.05), compared with control
Riva et al. (45)	Italy	*N* = 21 (M 8 and F 13; mean age 46.0 y) Condition: Dry eye Design: Randomized double blinded placebo-controlled trial Duration: 1 months	Anthocyanins (57.6 mg/d)	Tear secretion volume Pupil constriction	STT TriIRIS C9000	Significant improvement in tear secretion volume (*p* = 0.019), compared with placebo
Shim et al. (46)	Republic of Korea	N= 229 (M and F; mean age, 55.5 y) Condition: Normal tension glaucoma Design: Retrospective controlled trial Duration: mean duration 24.3 months	Anthocyanins (60 mg/d)	Visual acuity Visual field	BCVA HFA (30–2)	Significant improvement in BCVA (*p* = 0.008) and HFA mean deviation (*p* = 0.001) compared with control
Vetrugno et al. (47)	Italy	*N* = 97 (M 48 and F 49; mean age 65.3 y) Condition: Primary open angle glaucoma Design: Open, prospective, randomized, case–control trial Duration: 1 month	Rutin (200 mg/d) Forskolin (15 mg/d)	IOP	GAT	Significant decrease in IOP (*p* < 0.01), compared with control
Yamashita et al. (48)	Japan	*N* = 74 (M 16 and F 58; mean age 44.8 y) Condition: Dry eye and fatigue Design: Randomized double-blind placebo-controlled trial Duration: 1 month	Anthocyanins (21 mg/d) Delphinidins (19 mg/d)	Lacrimal fluid amount Tear film stability Eye fatigue level Eye fatigue and ophthalmic nerve sensitivity Eye-related subjective symptoms Visual acuity IOP	STT BUT Pupillary response Handy Flicker HF-II VAS DEQS Visual acuity test Tonometry	Significant higher lacrimal fluid production (*p* = 0.005), lower eye fatigue symptoms (*p* = 0.047), and bothersome ocular symptoms (*p* = 0.037), compared with placebo
Yoshida et al. (39)	Japan	*N* = 58 (M and F; mean age 62.3 y) Condition: Open angle glaucoma Design: Randomized double-blind placebo-controlled trial Duration: 24 months	Anthocyanins (50 mg/d)	Serum ET-1	ET-1 immunoassay	Increased levels of serum ET-1, compared with placebo (*p* < 0.05)

*ECGC, epigallocatechin-gallate; RGC, retinal ganglion cell; PERG, Pattern evoked electroretinograms; HFA, Humphrey field analyzer; OAG, open-angle glaucoma; BCVA, Best-corrected visual acuity; SD-OCT, Spectral domain-optical coherence tomography; MP, microperimetry; SLO scanning laser ophthalmoscope; HRA, Heidelberg retina angiograph; IOP, intraocular pressure; IOP_GAT_, intraocular pressure by Goldmann applanation tonometer; IOP_RBT_, intraocular pressure by rebound tonometry FA, Fundus photography; ERG, Electroretinogram; GAT, Goldmann applanation tonometer; ET-1, Endothelin-1; LDF, Laser Doppler Flowmeter; LSFG, Laser speckle flowgraphy; PSV, Peak systolic velocity; EDV, End diastolic volume; A, Acceleration; CRVRV, Central retinal vein reflux velocity; STT, Schirmer tear test; BUT, break-up time; VAS, Visual analog scale; DEQS, Dry eye-related quality of life score*.

### Flavonoids and Ocular Disorders

Seven of the 16 studies used a flavonoid alone (33–39), and the remaining used mixed supplements (40–48). The dose of flavonoids ranged from 19 milligrams to 300 milligrams per day. The duration of intervention also varied from 1 to 36 months. Various flavonoids were assessed in the studies included in this review. The most commonly tested groups of flavonoids were anthocyanins (35, 36, 39, 45, 46, 48); other subclasses of flavonoids included flavan-3-ols [e.g., epigallocatechin-gallate (ECGC) and proanthocyanidins] (33, 34, 42), isoflavones (e.g., puerarin and genistein) (38, 43), and flavonols (e.g., rutin and troxerutin) (40, 44, 47). None of the studies used flavanones. All studies examined the effect of flavonoid supplementation on the most common clinical outcomes associated with ocular conditions, such as visual acuity, retinal sensitivity and thickness, macular volume, ocular pressure, and tear secretion. An extensive number of diagnostic tests have been used in the included studies, such as best-corrected visual acuity (BCVA) assessment, electroretinogram, tonometry, and fundus photography. Seven studies involving 506 participants focused on glaucoma (33, 35, 36, 39, 42, 46, 47), and three clinical trials focused on DR (34, 38, 43). Two studies, authored by the same group, evaluated the different outcomes associated with cystoid macular edema (40, 41). Two trials explored the effect of flavonoids on dry eye (45, 48), and the last two studies involved participants with high intraocular pressure (IOP) (37, 44).

### Meta-Analyses

**Figure 2:**
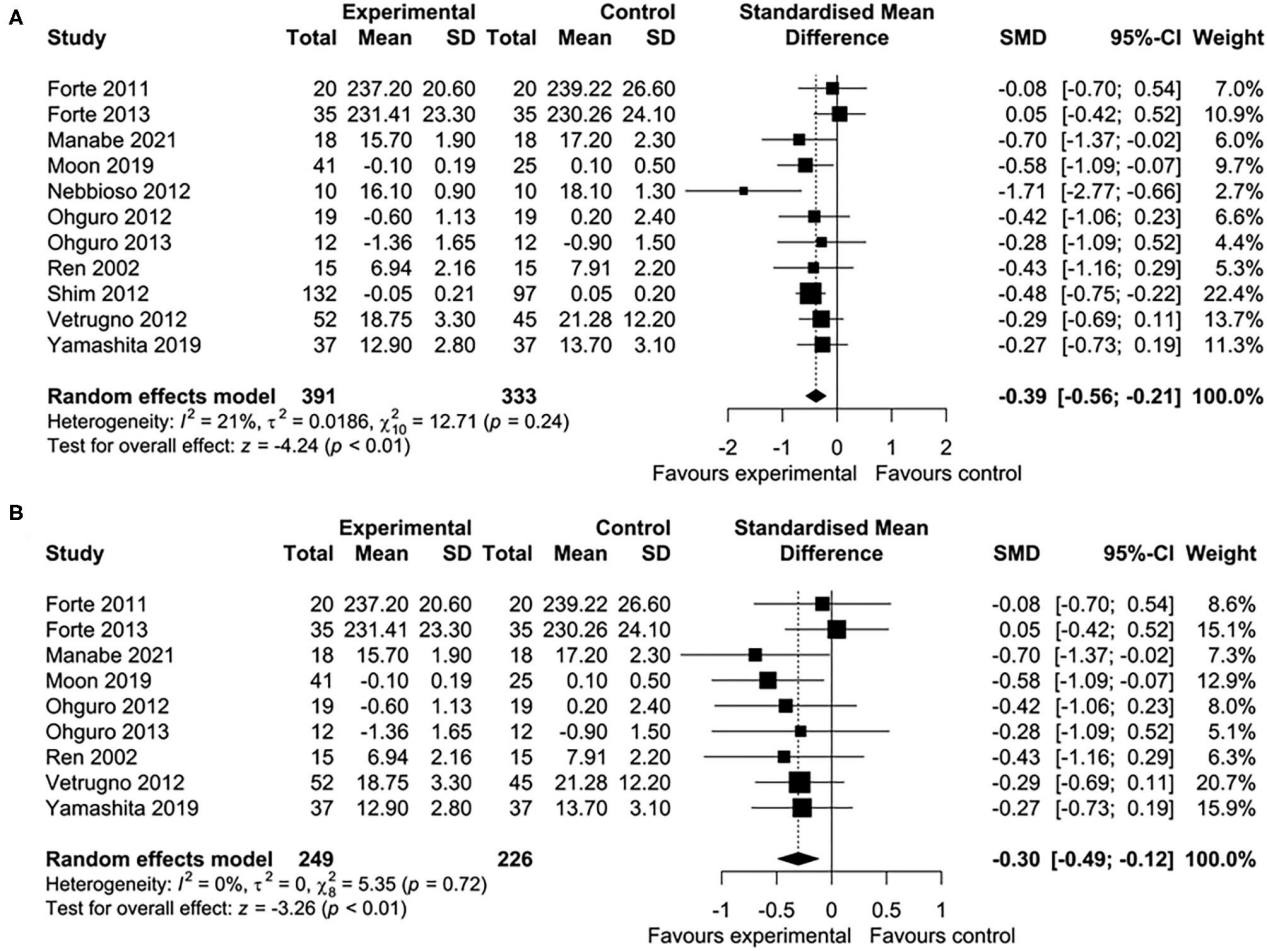
Forest plots showing the effect of flavonoid supplementation on ophthalmic conditions. **(A)** Forest plot showing the effect of flavonoids in 10 clinical trials on ocular disorders. **(B)** Sensitivity analysis summarizing the effect of flavonoid supplementation on ocular disorders.

**Figure 3:**
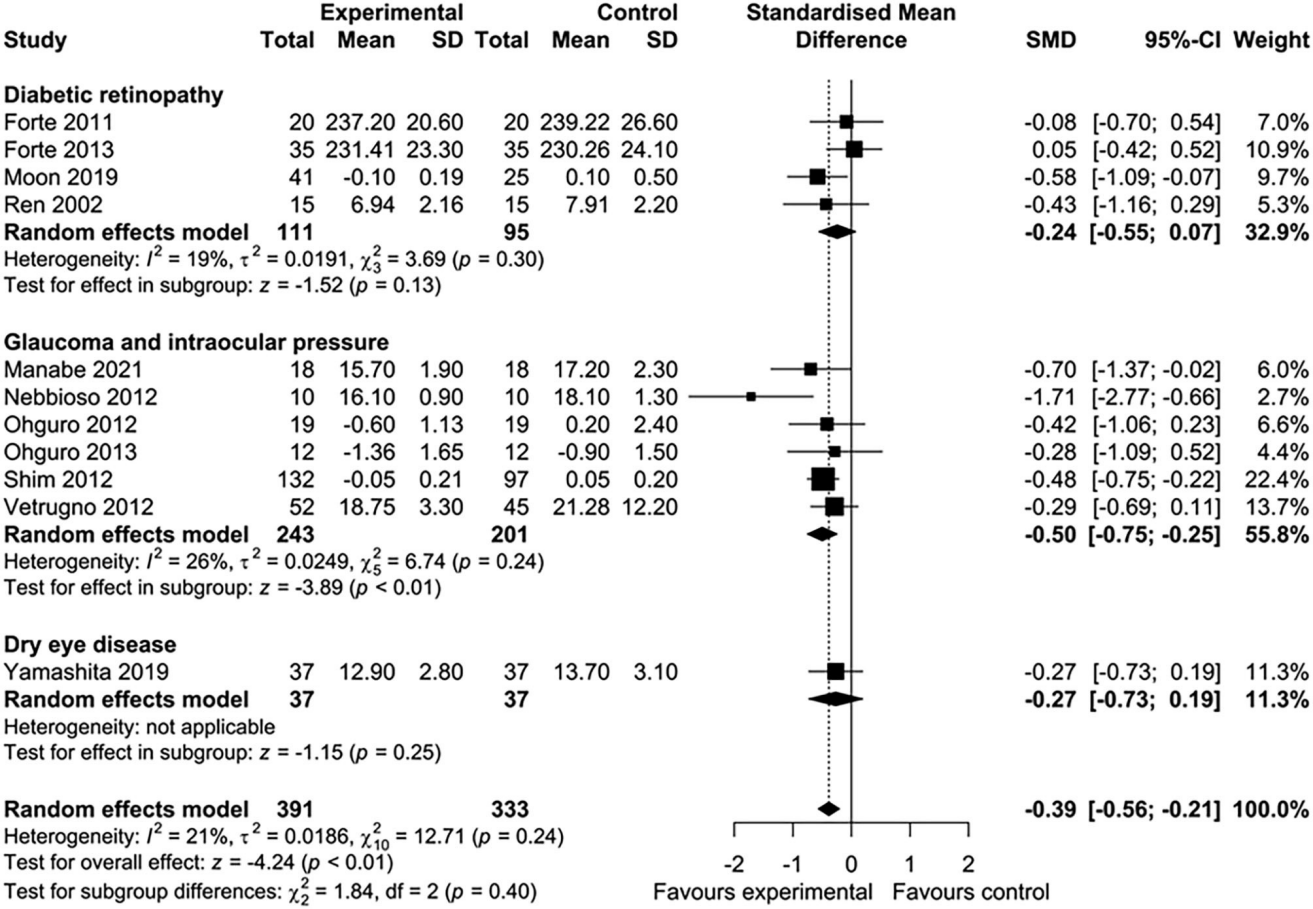
Meta-analysis to assess the effect of flavonoids in different subgroups of ocular disorders.

**Figure 4:**
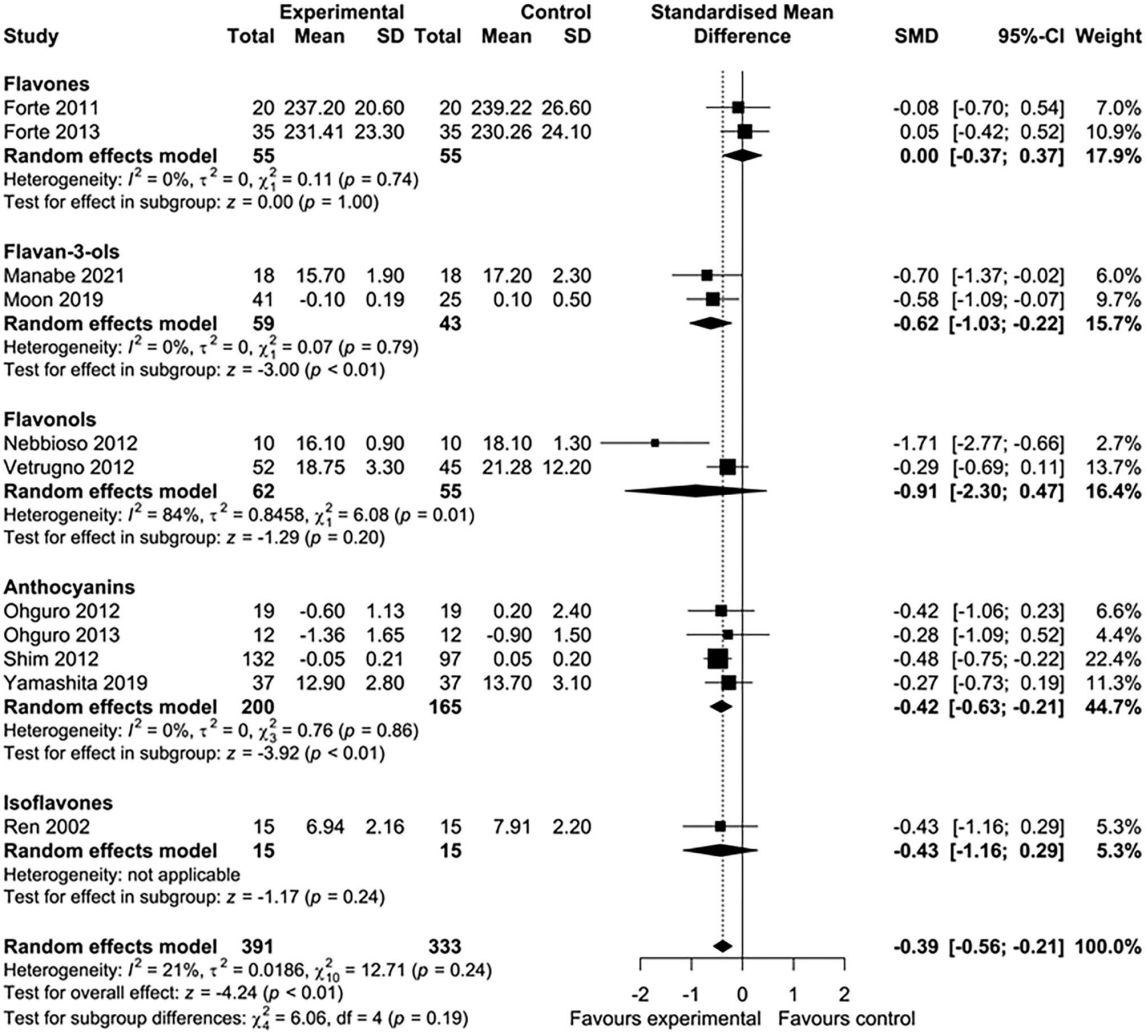
Subgroup analysis to assess the effect of flavonoid subclasses on ocular disorders.

Five of 16 studies were excluded from the meta-analysis because the required aggregate data for pooling were not available (33, 35, 39, 43, 45). In total, 11 studies involving 724 participants were included in this meta-analysis. Nutritional interventions with flavonoids showed an overall significant effect on various ophthalmic conditions, such as diabetic cystoid macular edema, DR, and gluacoma (standard mean difference = −0.39; 95% CI: −0.56, −0.21, *p* < 0.01) (Figure 2A). Although there was low heterogeneity among the included studies (*I*^2^ = 21%, τ^2^ = 0.0186, χ^2^ = 12.71, *p* = 0.24), we conducted a sensitivity analysis by one study at a time to observe the changes in the effects. After removing two studies (44, 46), the results remained essentially unchanged but with no evidence of heterogeneity (standard mean difference = −0.30; 95% CI: −0.49, −0.12, *p* < 0.01) (*I*^2^ = 0%, τ^2^ = 0, χ^2^ = 5.35, *p* = 0.72) (Figure 2B). The total number of participants included for the sensitivity analysis was 475. When the data were sufficient, we performed subgroup analyses based on ocular conditions and flavonoid subclasses. The results of the subgroup analysis of the effects of flavonoids on eye conditions are shown in Figure 3. We found a significant improvement in glaucoma and IOP after flavonoid supplementation (standard mean difference = −0.50; 95% CI: −0.75, −0.25, *p* < 0.01; *n* = 444 patients). However, there was a moderate evidence of heterogeneity among the studies (*I*^2^ = 37%). When subgroup analysis was conducted based on DR, no significant effect of flavonoids was observed (standard mean difference = −0.24; 95% CI: −0.55, −0.07, *p* = 0.13; *n* = 206 patients). The flavonoids were classified into subclasses for subgroup analysis (Figure 4). Although we pooled two clinical studies involving 102 patients, flavan-3-ol intervention showed a statistically overall effect (standard mean difference = −0.62; 95% CI: −1.03, −0.22, *p* < 0.01; *I*^2^ = 0%). The effect of anthocyanin supplementation was assessed in four clinical trials (*n* = 365 patients), and a significant overall effect was observed with no evidence of heterogeneity (standard mean difference = −0.42; 95% CI: −0.63, −0.21, *p* < 0.01; *I*^2^ = 0%). For several of the subclasses, however, there was no significant effect of flavonoids.

### Risk of Bias

The results of the risk of bias assessment are illustrated in Supplementary Figure 1, Supplementary Table 2. Although the most studies were randomized, several failed to describe the process involving sequence generation or allocation concealment in sufficient detail (33, 38–41, 43, 45, 47). Therefore, these studies were judged as having a high or an unclear risk of bias for these domains. Five studies were not randomized trials, and they were considered to have a high risk of bias (35, 37, 42, 44, 46). Seven trials did not have enough information on how participants and assessors were blinded (35, 38, 40, 41, 43, 46, 47). These studies were subsequently assigned a high risk of bias. All studies, except one trial (34), reported the number of dropouts during the interventions and provided adequate information about the original outcomes in the results section. Consequently, these studies were assigned low risks of bias in the domains associated with incomplete outcome data and selective reporting. Regarding the possibility of publication bias, visual inspection of the funnel plot revealed a symmetrical distribution of studies (Supplementary Figures 2A,B). Additionally, the Egger test (*p* = 0.50) and the Begg test (*p* = 0.31) did not show significant publication bias. Thus, the likelihood of publication bias was low.

## Discussion

To our knowledge, this is the first systematic review and meta-analysis to comprehensively summarize the effects of flavonoid supplementation on some of the most common ophthalmic disorders. Despite an extensive systematic search, we identified only 16 studies that met the eligibility criteria for inclusion in the systematic review. The overall quantitative analysis of 11 studies investigating a total of 724 participants showed that flavonoids may have a positive effect on eye conditions associated with visual dysfunction. An analysis, which was restricted to nine clinical studies, also found a significant improvement with no evidence of heterogeneity. Although flavonoids have been shown to improve the clinical outcomes of ocular disorders, these findings may not be generalizable. Minimal non-significant treatment effects were found for the subgroups of trials that tested the effect of flavonoids on DR and dry eye disease. Conversely, flavonoid compounds appear to have a greater effect on outcomes associated with glaucoma and IOP. These results are consistent with a previous meta-analysis that examined the effect of flavonoids on visual function in patients with glaucoma or ocular hypertension (49). However, the influence of flavonoids and their subclasses on ocular disorders has not been established. Firstly, most of the trials examined used different treatment durations and involved various dosages and sources of flavonoids. Secondly, only a few studies were meta-analyzed according to the type of flavonoids. Most of the flavonoid subclasses did not show any effect on eye disorders. However, when we pooled results from clinical trials that tested flavan-3-ols and anthocyanins, the overall effect was a statistically significant improvement in eye disorders.

Five studies could not be pooled in the meta-analysis, because they did not provide sufficient data to calculate the effect size. Despite this, the trial by Falsini et al. in patients with open-angle glaucoma treated with oral epigallocatechin-gallate (EGCG) suggests a beneficial influence of this compound on inner retinal function (33). Two other studies excluded from the meta-analysis showed that the flavonoid intervention had a statistically significant effect on clinical outcomes associated with glaucoma (35, 39). Likewise, patients affected by DR and dry eye disease also showed a significant improvement after the flavonoid intervention (43, 45).

The potential mechanisms underlying the reduction in the risk of eye diseases by flavonoids probably involve more than one pathway; these have been reported to be most often related to the antioxidant, anti-inflammatory, and anti-angiogenic functions (50). These mechanisms stabilize collagen, improve microvascular integrity, and, consequently, restore proper RPE function (51). Flavonoids can decrease oxidative damage through free radical scavenging activity. Moreover, they can upregulate endogenous antioxidant defense systems via signal transduction mechanisms at very low concentrations in retinal cells (52). High flavonoid intakes were associated with lower concentrations of biomarkers of inflammation, such as nuclear factor-kB (NF-kB) and C-reactive protein (CRP) (53). In addition, flavonoids modulate several transcription factors involved in angiogenesis, such as vascular endothelial factor (VEGF), basic fibroblast growth factor (bFGF), and hypoxia-inducible factor-1α (HIF-1α) (54). Another possible mechanism is that flavonoids modulate signaling pathways responsible for maintaining neuron survival (55).

It is important to note that the beneficial effects of flavonoids may depend on their bioavailability, which differs greatly with the subclass. In the case of the eye, flavonoids need be transported from the gastrointestinal tract to the circulatory system and across the blood-retinal barrier, including the retinal vascular endothelium and retinal pigment epithelium. Some studies reported that flavonoids are bioavailable to the eye in quantities that can affect signal transduction mechanisms and influence enzymatic functions within the eye (56). For example, anthocyanins were detected in tissues beyond the blood-brain barrier (57). This may account, at least in part, for the positive effects of anthocyanins observed in this meta-analysis.

## Limitations

Some limitations should be acknowledged in the current meta-analysis. The outcomes of the present systematic review and meta-analysis are based on a relatively few studies. Therefore, the findings should be interpreted with caution. While most studies were of low and moderate risks of bias, the studies with a high risk of bias were also included in the analysis, which could have influenced the results. No studies measured the urinary concentrations of flavonoids to assess their absorption. Flavonoids and their subclasses are commonly consumed as part of a normal diet, and most of the studies did not measure the exposure to flavonoids using food frequency questionnaires. Therefore, we could not rule out bias due to the misclassification of dietary exposure. It should also be noted that the nature of the interventions differed across the studies; four included trials of flavonoids combined with other phytochemicals. Geographical restrictions were present in this meta-analysis, as all the original studies were from Europe (mostly Italy) and Asia. Therefore, our findings should be generalized to other ethnic groups with caution.

## Conclusions

In summary, the current systematic review and meta-analysis demonstrated that flavonoids have a favorable effect on conditions associated with visual impairment. The relatively low cost, the lack of serious side effects, and easy accessibility make flavonoid supplementation appealing. Our findings may provide an impetus to conduct more well-powered and high-quality clinical trials. Further studies are needed to better determine the optimal dosage, treatment duration, and the role of different flavonoid subclasses. It is also crucial to explore the long-term sustenance of improvements. If confirmed, these findings may have important implications for the preservation of eye health.

## Data Availability Statement

The raw data supporting the conclusions of this article will be made available by the authors, without undue reservation.

## Author Contributions

SD and CC conceived the presented idea and designed the study. SD and SA collected and analyzed data. SD and GS wrote the manuscript. CC and GS edited and reviewed the manuscript. All authors discussed the results, contributed to the final manuscript, approved the final version of the manuscript, and agree to be accountable for the study.

## Conflict of Interest

The authors declare that the research was conducted in the absence of any commercial or financial relationships that could be construed as a potential conflict of interest.
