# Supplementary material for: Effects of Flavonoid Supplementation on Common Eye Disorders: A Systematic Review and Meta-Analysis of Clinical Trials
Source: Front Nutr. 2021 May 25;8:651441. doi: 10.3389/fnut.2021.651441 (PMC8189261; doi:10.3389/fnut.2021.651441)

Effects of flavonoid supplementation on common eye disorders: A systematic review and meta-analysis of clinical trials

**Supplementary Material**

**Supplementary Table 1**

Search strategy used PubMed, Web of Science, Scopus, and Cochrane databases

| **Database** |  | **Type of search** |  | **Search terms** |
| --- | --- | --- | --- | --- |
|  |  |  |  |  |
| PubMed |  | General terms and Medical Subject Headings (MeSH) |  | "flavonoid" OR "anthocyanin" OR "anthocyanidin" OR "cyanidin" OR "delphinidin" OR "malvidin" OR "pelargonidin" OR "peonidin" OR "petunidin" OR "flavan-3-ol" OR "catechin" OR "epicatechin" OR "epigallocatechin" OR "gallocatechin" OR "proanthocyanidin" OR "theaflavin" OR "thearubigin" OR "flavonol" OR "isorhamnetin" OR "kaempferol" OR "myricetin" OR "quercetin" OR "flavone" OR "apigenin" OR "luteolin" OR "baicalein" OR "chrysin" OR "flavanone" OR "eriodictyol" OR "hesperetin" OR "naringenin" OR "isoflavone" OR "daidzein" OR "genistein" OR "glycitein" OR "biochanin" OR "formononetin" AND "ocular disease" OR "eye disorders" OR "eye diseases" OR "age-related ocular diseases" OR "age-related macular degeneration" OR "macular degeneration" OR "glaucoma" OR "cataract" OR "diabetic retinopathy" OR "presbyopia" OR "dry eye" NOT "animal" OR "in vivo" OR "in vitro" OR "rat" OR "mouse" OR "mice" OR "nonhuman" OR "cell line".  (flavonoids[MeSH Terms]) AND (eye diseases) [MeSH Terms]) AND (age-related eye diseases[MeSH Terms]) |
|  |  |  |  |  |
| Web of Science |  | General terms |  | "flavonoid" OR "anthocyanin" OR "anthocyanidin" OR "cyanidin" OR "delphinidin" OR "malvidin" OR "pelargonidin" OR "peonidin" OR "petunidin" OR "flavan-3-ol" OR "catechin" OR "epicatechin" OR "epigallocatechin" OR "gallocatechin" OR "proanthocyanidin" OR "theaflavin" OR "thearubigin" OR "flavonol" OR "isorhamnetin" OR "kaempferol" OR "myricetin" OR "quercetin" OR "flavone" OR "apigenin" OR "luteolin" OR "baicalein" OR "chrysin" OR "flavanone" OR "eriodictyol" OR "hesperetin" OR "naringenin" OR "isoflavone" OR "daidzein" OR "genistein" OR "glycitein" OR "biochanin" OR "formononetin" AND "ocular disease" OR "eye disorders" OR "eye diseases" OR "age-related ocular diseases" OR "age-related macular degeneration" OR "macular degeneration" OR "glaucoma" OR "cataract" OR "diabetic retinopathy" OR "presbyopia" OR "dry eye" NOT "animal" OR "in vivo" OR "in vitro" OR "rat" OR "mouse" OR "mice" OR "nonhuman" OR "cell line". |
|  |  |  |  |  |
| Scopus |  | General terms and INDEXTERMS |  | "flavonoid" OR "anthocyanin" OR "anthocyanidin" OR "cyanidin" OR "delphinidin" OR "malvidin" OR "pelargonidin" OR "peonidin" OR "petunidin" OR "flavan-3-ol" OR "catechin" OR "epicatechin" OR "epigallocatechin" OR "gallocatechin" OR "proanthocyanidin" OR "theaflavin" OR "thearubigin" OR "flavonol" OR "isorhamnetin" OR "kaempferol" OR "myricetin" OR "quercetin" OR "flavone" OR "apigenin" OR "luteolin" OR "baicalein" OR "chrysin" OR "flavanone" OR "eriodictyol" OR "hesperetin" OR "naringenin" OR "isoflavone" OR "daidzein" OR "genistein" OR "glycitein" OR "biochanin" OR "formononetin" AND "ocular disease" OR "eye disorders" OR "eye diseases" OR "age-related ocular diseases" OR "age-related macular degeneration" OR "macular degeneration" OR "glaucoma" OR "cataract" OR "diabetic retinopathy" OR "presbyopia" OR "dry eye" NOT "animal" OR "in vivo" OR "in vitro" OR "rat" OR "mouse" OR "mice" OR "nonhuman" OR "cell line".  (INDEXTERMS ("flavonoids") ) AND ( INDEXTERMS ( "eye diseases" ) ) |
|  |  |  |  |  |
| Cochrane Library |  | General terms and Medical Subject Headings (MeSH) |  | "flavonoid" OR "anthocyanin" OR "anthocyanidin" OR "cyanidin" OR "delphinidin" OR "malvidin" OR "pelargonidin" OR "peonidin" OR "petunidin" OR "flavan-3-ol" OR "catechin" OR "epicatechin" OR "epigallocatechin" OR "gallocatechin" OR "proanthocyanidin" OR "theaflavin" OR "thearubigin" OR "flavonol" OR "isorhamnetin" OR "kaempferol" OR "myricetin" OR "quercetin" OR "flavone" OR "apigenin" OR "luteolin" OR "baicalein" OR "chrysin" OR "flavanone" OR "eriodictyol" OR "hesperetin" OR "naringenin" OR "isoflavone" OR "daidzein" OR "genistein" OR "glycitein" OR "biochanin" OR "formononetin" AND "ocular disease" OR "eye disorders" OR "eye diseases" OR "age-related ocular diseases" OR "age-related macular degeneration" OR "macular degeneration" OR "glaucoma" OR "cataract" OR "diabetic retinopathy" OR "presbyopia" OR "dry eye" NOT "animal" OR "in vivo" OR "in vitro" OR "rat" OR "mouse" OR "mice" OR "nonhuman" OR "cell line".  #1 MeSH descriptor: [Flavonoids] explode all trees  #2 MeSH descriptor: [Eye Diseases] explode all trees  #1 and #2 |

**Supplementary Figure 1**.

Risk of bias assessment for the included clinical trials.

Risk of bias graph


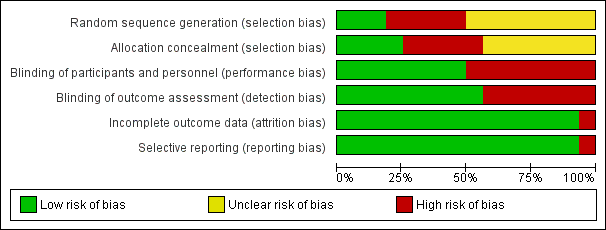


**Supplementary Table 2.**

Quality assessment of included clinical trials using he Cochrane Risk of Bias Tool

**Supplementary Figure 2**.

Funnel plots of clinical trials included in the meta-analysis.

(A) Funnel plot for overall studies included in the meta-analysis.


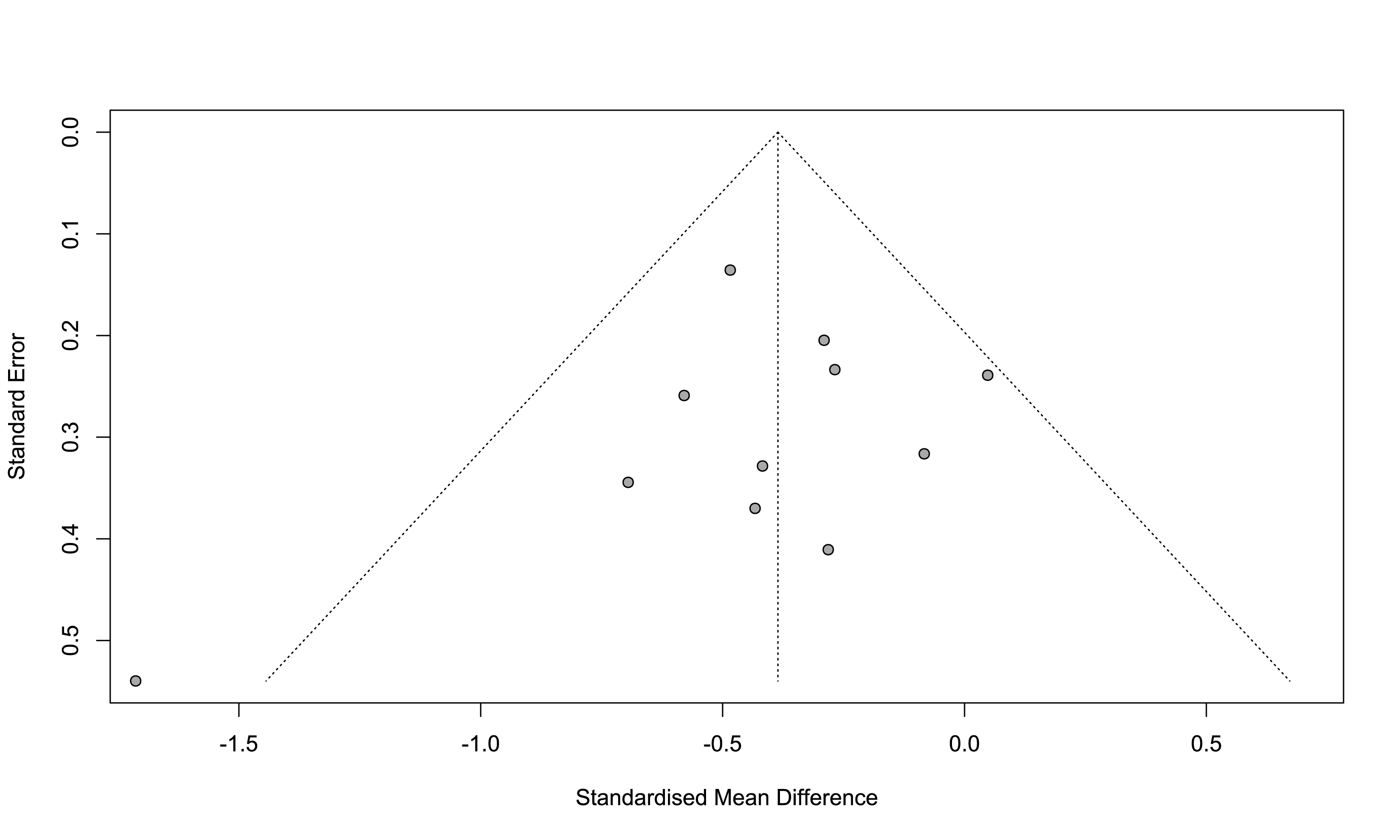


(B) Funnel plot for clinical trials after removing studies with potential publication bias.


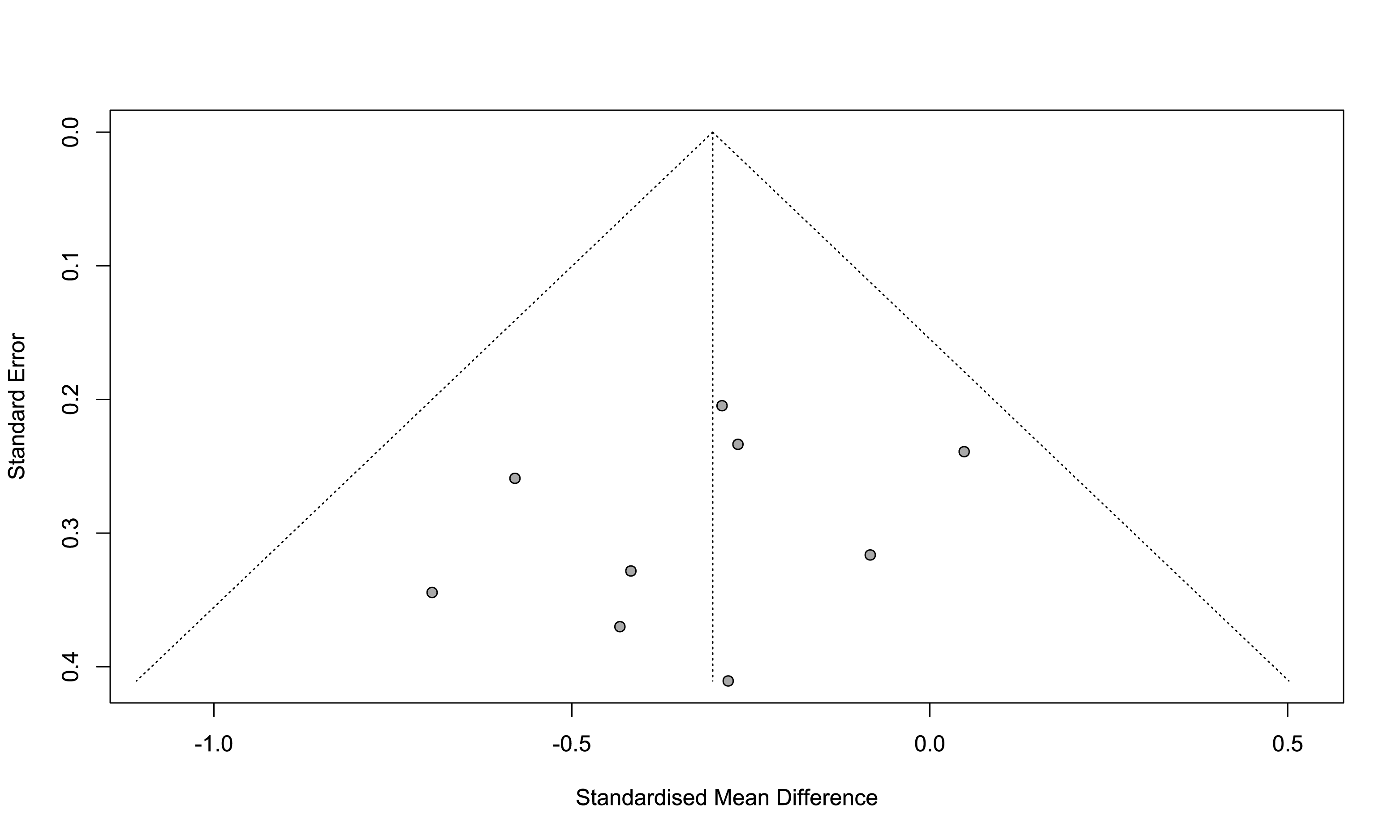

Supplement: Supplementary file 1 [file Data_Sheet_1.docx]
